# Supplementary material for: Exploring Treatment for Depression in Parkinson’s Patients: A Cross-Sectional Analysis
Source: Int J Environ Res Public Health. 2021 Aug 14;18(16):8596. doi: 10.3390/ijerph18168596 (PMC8392211; doi:10.3390/ijerph18168596)
Supplement: Supplementary file 1 [file ijerph-18-08596-s001.zip › ijerph-1297270-SI.pdf]

Supplemental Materials:

Supplemental Table S1. Pharmacologic Therapy for Depression

Supplemental Table S2. Anti-Parkinsonian Medication

Supplemental Table S1. Pharmacologic Therapy for Depression

NHANES Code	Drug
**c00249**	Antidepressants - Unspecified
**d08125**	Vortioxetine
**d00876**	Clomipramine
**d05355**	Duloxetine
**d03181**	Venlafaxine
**d08114**	Levomilnacipran
**d00061**	Lithium
**d03157**	Paroxetine
**d00236**	Fluoxetine
**d04917**	Fluoxetine; Olanzapine
**d04025**	Mirtazapine
**d08373**	Brexpiprazole
**d04812**	Escitalopram
**d07740**	Vilazodone
**d00168**	Alprazolam
**d00146**	Amitriptyline
**d03462**	Amitriptyline; Chlordiazepoxide
**d03463**	Amitriptyline; Perphenazine
**d00874**	Amoxapine
**d00803**	Amphetamine
**d04035**	Amphetamine; Dextroamphetamine
**d00182**	Buspirone
**d00189**	Chlordiazepoxide
**d03492**	Chlordiazepoxide; Clidinium
**h00012**	Chlordiazepoxide; Esterified Estrogens
**d05416**	Chlordiazepoxide; Methscopolamine
**d04332**	Citalopram
**d00145**	Desipramine
**d00217**	Doxepin
**882**	Isocarboxazid
**d00877**	Maprotiline
**d03808**	Nefazodone
**d00855**	Perphenazine
**d00137**	Pindolol
**d00875**	Protriptyline
**d00873**	Trimipramine

Supplemental Table S2. Anti-Parkinsonian Medication

NHANES Code	Drug
**d00184**	Carbidopa
**d04877**	Carbidopa; Entacapone; Levodopa
**d03473**	Carbidopa; Levodopa
**d04537**	Rivastigmine
**d00086**	Amantadine
**h00026**	Acetaminophen; Amantadine; Chlorpheniramine
**h00031**	Acetaminophen; Amantadine; Chlorpheniramine; Phenylephrine
**d04145**	Pramipexole
**d05848**	Rotigotine
**d04215**	Ropinirole
**d00976**	Selegiline
**d04991**	Apomorphine
**d00178**	Bromocriptine
**d04460**	Entacapone
**d00277**	Levodopa
**d04750**	Galantamine
**d04220**	Quetiapine
**d05612**	Rasagiline
